# Magnetic resonance guided focused ultrasound capsulotomy for intractable obsessive compulsive disorders

**DOI:** 10.1186/2050-5736-3-S1-O8

**Published:** 2015-06-30

**Authors:** Jin Woo Chang

**Affiliations:** 1 Yonsei University College of Medicine, Seoul, Republic of Korea

## Background/introduction

Surgery for intractable psychiatric illness has generated considerable controversy for a variety of scientific, social and philosophical reasons. However, the surgical treatment of obsessive compulsive disorders (OCD) by lesioning techniques such as cingulotomy, capsulotomy was well accepted in the clinical field throughout the world. However, the lacks of direct neuroanatomical and pathophysiological rationales for how lesions in specific limbic areas alleviate specific OCD symptoms have been consistent criticisms of lesioning procedures. Recently, because the anatomical and neurochemical substrates of brain function in health controls and disease patients are slowly being elucidated by various functional neuroimaging techniques, these criticisms are becoming less valid. Furthermore, by using new technique such as deep brain stimulation (DBS) and by making more precise targets, it enables to treat the patients without making serious complications.

## Methods

However, as we recognize, DBS also has many disadvantages along with procedures and etc. Currently, MR-guided focused ultrasound (MRgFUS) has been developed as a non-invasive surgical tool of generating precisely placed focal thermal lesion in the brain. The authors underwent a feasibility study of MRgFUS for the treatment of medically refractory OCD. Patients with OCD were treated by making bilateral thermal lesions in the anterior limb of the internal capsule (capsulotomy) with MRgFUS.

In this presentation, I would like to demonstrate not only the therapeutic effects but also technical & practical issues of the current MRgFUS for medically refractory OCD.

